# P13 Predictive value of urinalysis and urine culture in bacteraemic adults without localizing urinary features: a retrospective cohort study

**DOI:** 10.1093/jacamr/dlad077.017

**Published:** 2023-08-02

**Authors:** Chris Hopkins, Jenna Beaumont, Nicholas Young

**Affiliations:** Middlemore Hospital, Auckland, New Zealand; Auckland Hospital, Auckland, New Zealand; Auckland Hospital, Auckland, New Zealand

## Abstract

**Background:**

Urinalysis (UA) and urine culture (UC) are commonly used in patients with bacteraemia to diagnose a urinary focus of infection. The predictive value of these investigations in patients without localizing urinary features is uncertain. We hypothesized that UA/UC would have extremely low predictive value in patients with bacteraemia and neither localizing urinary features nor risk factors for bacteraemic UTI (bUTI).

**Methods:**

This was a retrospective cohort study of hospitalized adults with community-onset bacteraemia in 2019 at Middlemore Hospital (Auckland, New Zealand). The suspected focus of infection at first assessment was determined by reviewing clinical notes from the initial presentation using the admitting clinician’s preferred diagnosis plus at least one supportive clinical feature. The final diagnosis of focus of bacteraemia (bUTI versus non-urinary) was determined by investigators based on blood and urine culture concordance, with supportive clinical, laboratory or imaging features. Defined localizing urinary features were determined by investigators from notes. Positive and negative predictive value of UA/UC for diagnosing bUTI was calculated among those with initially unclear or non-urinary focus of infection. Univariate analysis was performed for patients with initially unclear focus of infection, for risk factors associated with final diagnosis of bUTI.

**Results:**

In total, 871 patients were included. The admitting clinician’s suspected focus was urinary in 219, unclear in 304 and non-urinary in 348. A final diagnosis of bUTI was found in 342 (39.3%), of whom 110 (32.2%) lacked any localizing urinary features. After excluding localizing urinary features, bUTI was diagnosed in 98/243 (40.3%) with initially unclear focus and 12/326 (3.7%) with suspected non-urinary focus. UA/UC PPV was 5.2%/25%, respectively, when initial focus was non-urinary, but 51.5%/75% when initial focus was unclear. Amongst patients with initially unclear focus, risk factors for bUTI included age >75, urinary catheter/nephrostomy, invasive urologic procedure within 3 months and cognitive impairment. However, of those with bUTI, 22/98 (22.4%) had none of these risk factors.

**Conclusions:**

bUTI occurs in a significant minority of bacteraemic patients where initial focus is unclear and where localizing urinary features are absent, including in patients without associated risk factors. UA/UC is a reasonable investigation in bacteraemia of uncertain focus, but results should be interpreted cautiously. UA has excellent NPV but very poor PPV. Among patients with bacteraemia, initially suspected non-urinary focus of infection, and without localizing urinary features, UA/UC can be safely withheld and bUTI is very uncommon. A prospective study using initial clinical criteria would provide more relevant predictive values for clinicians.

